# The 2021 World Health Organization classification of gliomas: an imaging approach

**DOI:** 10.1590/0100-3984.2022.0089-en

**Published:** 2023

**Authors:** Renata Tarraf Fernandes, Gustavo Ramos Teixeira, Esther Cecin Mamere, Gabriela Alencar Bandeira, Augusto Elias Mamere

**Affiliations:** 1 Hospital de Câncer de Barretos, Barretos, SP, Brazil; 2 Universidade Federal do Rio Grande do Norte (UFRN), Natal, RN, Brazil; 3 Instituto de Radiologia do Hospital das Clínicas da Faculdade de Medicina da Universidade de São Paulo (InRad/HC-FMUSP), São Paulo, SP, Brazil

**Keywords:** Glioma/classification, Central nervous system neoplasms/classification, Glioblastoma/classification, Astrocytoma/classification, Gliomas/classificação, Neoplasias do sistema nervoso central/classificação, Glioblastomas/classificação, Astrocitomas/classificação

## Abstract

The purpose of this pictorial essay is to describe the recommendations of the 2021 World Health Organization classification for adult-type and pediatric-type gliomas and to discuss the main modifications in relation to the previous (2016) classification, exemplified by imaging, histological, and molecular findings in nine patients followed at our institutions. In recent years, molecular biomarkers have gained importance in the diagnosis and classification of gliomas, mainly because they have been shown to correlate with the biological behavior and prognosis of such tumors. It is important for neuroradiologists to familiarize themselves with this new classification of central nervous system tumors, so that they can use this knowledge in evaluating and reporting the imaging examinations of patients with glioma.

## Abstract (pt)

O propósito deste ensaio iconográfico é descrever e discutir as novas recomendações da Organização Mundial da Saúde de 2021, referente aos gliomas dos tipos adulto e infantil, e suas principais diferenças com a classificação anterior (2016), exemplificadas com imagens de nove casos de pacientes atendidos nas nossas instituições. Recentemente, há uma crescente significância dos marcadores moleculares no diagnóstico e classificação dos gliomas e tumores do sistema nervoso central, principalmente pela correlação com o comportamento biológico e o prognóstico. É importante que os neurorradiologistas estejam familiarizados com a nova classificação dos tumores do sistema nervoso central para a prática clínica, na avaliação e emissão de laudos e opiniões nas imagens dos pacientes com gliomas.

## INTRODUCTION

In recent years, molecular markers have gained importance in the diagnosis and classification of gliomas, mainly because they have been shown to predict the biological behavior and prognosis of these tumors. In its 2016 classification, the World Health Organization (WHO) recommended that evaluation of molecular markers be incorporated into the investigation of certain central nervous system tumors^(1)^. In the most recent (2021) WHO classification, that recommendation was expanded to include new biomarkers^(2,3)^. For gliomas, the main differences were the division between adult-type and pediatric-type gliomas; the combination of histological and molecular findings in the classification of glial neoplasms; the recognition of new neoplastic entities; and the revision of the nomenclature, including the abolition of grading terms such as anaplastic. Adult-type and pediatric-type gliomas are expected to occur more commonly, although not exclusively, in their respective age groups. Nevertheless, adult-type gliomas rarely affect pediatric patients, and vice versa. In addition, the 2021 WHO classification abolished the use of Roman numerals in the histological grading of tumors, recommending the use of Arabic numerals in order to avoid confusion between grades II and III, especially because this new classification also abolished the use of some terms modifying histological grading^(3)^.

The primary objective of this pictorial essay is to describe, through the use of the imaging, histological, and molecular findings of nine cases, the new recommendations of the 2021 WHO classification regarding glial neoplasms in pediatric and adult patients. A secondary objective is to compare and contrast the 2016 and 2021 classifications.

## ADULT-TYPE DIFFUSE GLIOMAS

The new (2021) WHO classification includes only three categories of adult-type diffuse gliomas: isocitrate dehydrogenase (IDH)-mutant astrocytoma; IDH-mutant, 1p/19q-codeleted oligodendroglioma; and IDH wild-type glioblastoma.

### IDH-mutant astrocytomas

**Figure 1:**
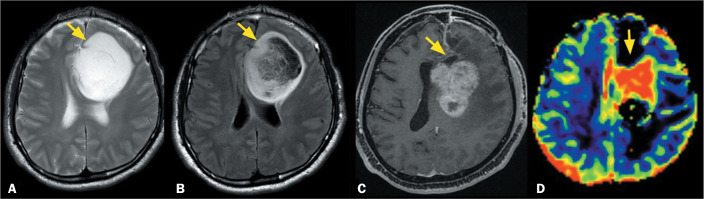
MRI scans of a 25-year-old male patient. A T2-weighted sequence (A) and a FLAIR sequence (B), both acquired in 2010, showing an infiltrative lesion, with high signal intensity and a T2-FLAIR mismatch sign (arrows), in the left frontal lobe. After surgical resection, the patient received a diagnosis of IDH-mutant grade 2 astrocytoma. A follow-up examination in 2021 (11 years later) showed enlargement of the remaining lesion. At that time, a fat-saturated T1-weighted sequence (C) showed areas of intense contrast enhancement in the solid areas (arrow) and a dynamic T2* perfusion-weighted sequence (D) showed high rCBV (arrow on color map), both findings being consistent with transformation to high grade. After a second surgical resection, a diagnosis of IDH-mutant grade 4 astrocytoma was made. No 1p/19q codeletion was observed.

IDH-mutant astrocytomas are classified, according to their histological characteristics, as grade 2 (low grade) or as grade 3 or 4 (high grade). Low-grade (grade 2) astrocytomas are slow-growing, infiltrative, poorly defined tumors, with thickening of the gyri and high signal intensity on T2-weighted magnetic resonance imaging (MRI) sequences. Typically, they do not show contrast enhancement and have low relative cerebral blood volume (rCBV) on dynamic T2* perfusion-weighted MRI sequences. Some show the T2-fluid-attenuated inversion recovery (FLAIR) mismatch sign^(4)^, which is characteristic of, although not exclusive to, this histological type. High-grade (grade 3 and 4) astrocytomas can show areas of contrast enhancement and high rCBV. In grade 4 astrocytomas, areas of central necrosis can be seen. The presence of a CDKN2A/B homozygous deletion is indicative of a high-grade astrocytoma, even in the absence of high-grade histological findings, such as microvascular proliferation and necrosis. Low-grade IDH-mutant astrocytomas can undergo late transformation to high grade (Figure 1).

### IDH-mutant, 1p/19q-codeleted oligodendrogliomas

**Figure 2:**
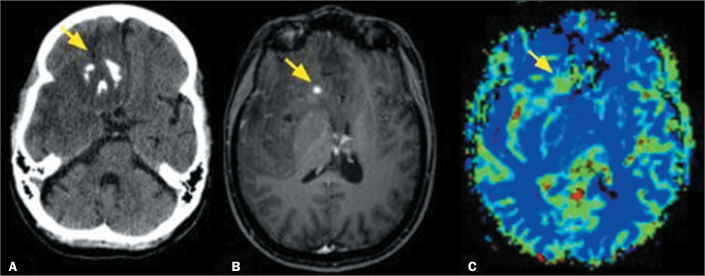
A 26-year-old female patient. A computed tomography scan (A) and MRI scans (B,C), showing an infiltrative, poorly defined lesion, containing gross calcifications (arrow in A), in the right cerebral hemisphere. Gadolinium contrast-enhanced, fat-saturated T1-weighted sequence showing a small area of contrast enhancement within the lesion (arrow in B) and a dynamic T2* perfusion-weighted sequence (C) showing a high rCBV (arrow on color map). The results of the histological and molecular analyses were consistent with a diagnosis of IDH-mutant, 1p/19q-codeleted grade 2 oligodendroglioma.

The molecular characteristic that defines oligodendrogliomas is the loss of the short arm of chromosome 1 (1p) and of the long arm of chromosome 19 (19q), characterizing the 1p/19q codeletion, which is associated with an IDH mutation^(3)^. Oligodendrogliomas are categorized as low-grade (grade 2) or high-grade (grade 3) neoplasms, according to their histological characteristics. On radiological imaging, these tumors appear as infiltrative lesions quite similar to astrocytomas, with one distinction: the presence of possible gross calcifications, which can be seen on computed tomography, often following a gyriform pattern. As illustrated in Figure 2, small foci of enhancement and increased rCBV on perfusion-weighted sequences are considered acceptable in such cases and do not represent high-grade transformation, as they would for astrocytomas.

### IDH wild-type glioblastomas

**Figure 3:**
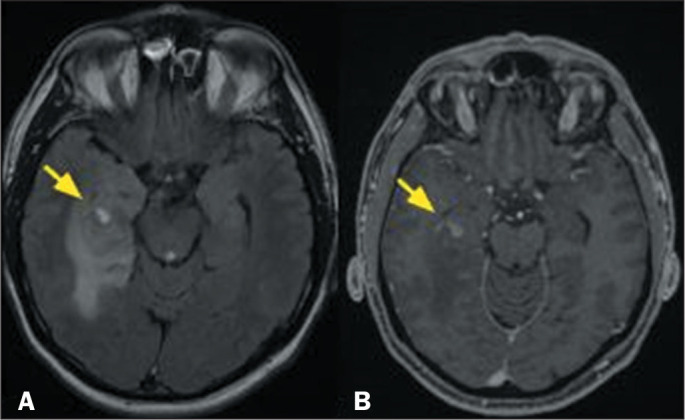
MRI scans of a 53-year-old male patient. A FLAIR sequence (A) showing an infiltrative lesion with high signal intensity in the right temporal lobe, together with an area of necrosis/liquefaction (arrow), and a gadolinium contrast-enhanced, fat-saturated T1-weighted sequence (B) showing a small focus of contrast enhancement (arrow), both findings being suggestive of a high-grade lesion. The histological findings were consistent with a diagnosis of low-grade (grade 2) astrocytoma. However, the molecular study showed IDH wild-type, mutation of the TERT promoter gene, and amplification of the EGFR gene, consistent with a diagnosis of molecularly defined glioblastoma, according to the latest (2021) WHO classification.

Glioblastomas are diffuse, IDH wild-type astrocytic gliomas with at least one of the following histological or molecular features^(3)^: microvascular proliferation; necrosis; telomerase reverse transcriptase (TERT) promoter mutation; amplification of the epidermal growth factor receptor (EGFR) gene; and gain of chromosome 7 and loss of chromosome 10. It is possible to make a diagnosis of glioblastoma on the basis of molecular markers alone (mutation of the TERT promoter, amplification of the EGFR gene, and gain of chromosome 7/loss of chromosome 10) even without high-grade histological findings (necrosis and microvascular proliferation), in which case it is considered molecularly defined glioblastoma (Figure 3).

## PEDIATRIC-TYPE DIFFUSE GLIOMAS

In the 2021 WHO classification, pediatric-type diffuse gliomas are divided into low and high grade, which differ significantly in terms of the clinical approach, surgical management, and prognosis.

### Pediatric-type diffuse low-grade gliomas

The 2021 WHO classification includes four entities in the group of pediatric-type diffuse low-grade gliomas^(3)^: MYB/MYBL1-altered diffuse astrocytoma; angiocentric glioma; polymorphous low-grade neuroepithelial tumor of the young; and mitogen-activated protein kinase pathway-altered diffuse low-grade glioma. The imaging characteristics of these tumors are similar to those of low-grade astrocytomas in adults, being poorly defined and infiltrative, with thickening of the gyri and high signal intensity on T2-weighted and FLAIR sequences, with or without minimal contrast enhancement, and presenting slow growth, which, in some cases, results in bone remodeling in the cranial vault.

**Figure 4:**
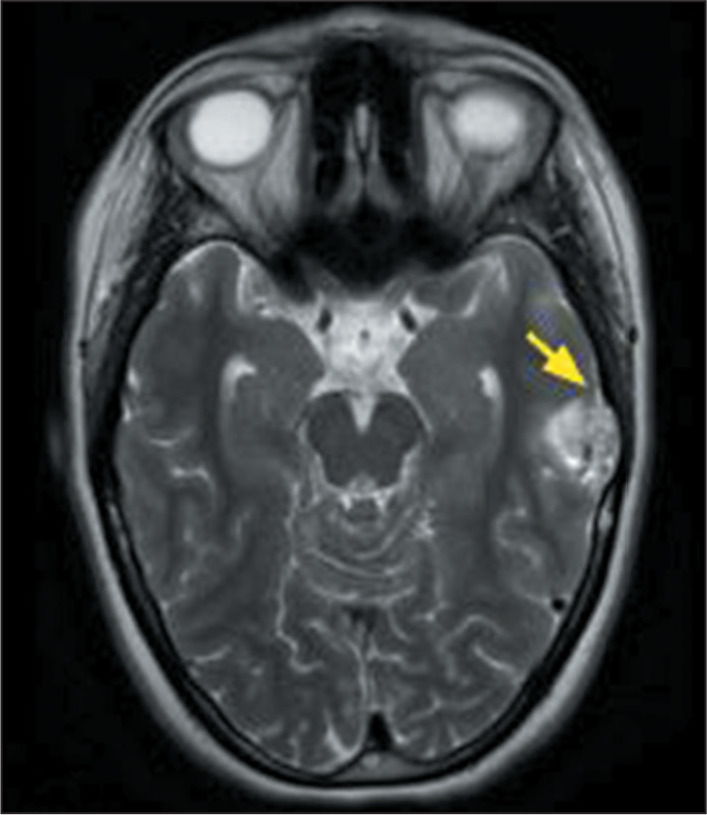
MRI examination of a 20-year-old female patient diagnosed with a polymorphous low-grade neuroepithelial tumor of the young. A T2-weighted sequence showing a well-defined intra-axial temporal lesion on the left, with high signal intensity and containing small cystic areas, the slow growth of which led to remodeling of the cranial vault (arrow).

Polymorphous low-grade neuroepithelial tumors of the young (Figure 4) have some distinct characteristics^(5)^: infiltrative growth; being more well defined than other pediatric-type diffuse low-grade gliomas; potentially presenting intratumoral cysts with a peripheral distribution; and often evolving to gross central calcifications, which can be seen on computed tomography. Those features allow the differential diagnosis with adult-type, IDH-mutant 1p/19q-codeleted oligodendroglioma.

**Figure 5:**
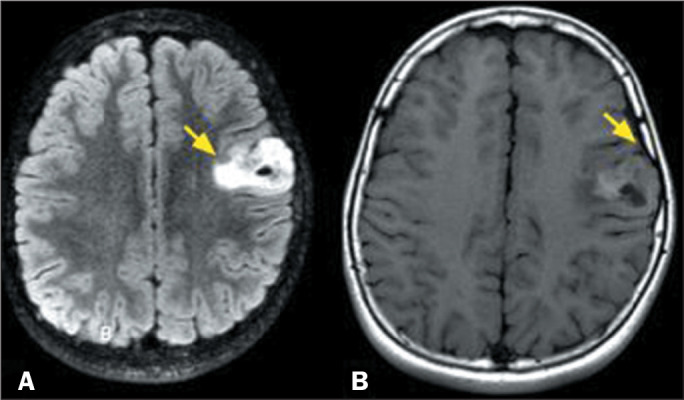
MRI scans of a 16-year-old male patient. A FLAIR sequence (A) showing an infiltrative lesion in the left frontal lobe (arrow) and a T1-weighted sequence (B) showing areas of high signal intensity. The lesion showed slow growth, which resulted in remodeling of the adjacent cranial vault (arrow in B). The histological findings were consistent with a diagnosis of angiocentric glioma.

Having only recently been described, angiocentric gliomas (Figure 5) are slow-growing, well-defined tumors, with high signal intensity on T2-weighted and FLAIR sequences, which can form small cysts. In some cases, angiocentric gliomas show a small area of signal alteration, extending from the tumor to the ependymal surface, and an atrophic aspect, mimicking encephalomalacia, or even peripheral areas with high signal intensity on T1-weighted sequences^(6-8)^.

### Pediatric-type diffuse high-grade gliomas

**Figure 6:**
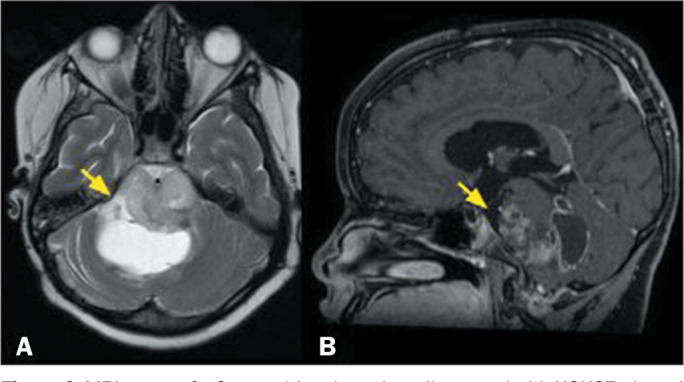
MRI scans of a 9-year-old male patient diagnosed with H3K27-altered diffuse midline glioma. A T2-weighted sequence (A) showing an expansile, infiltrative lesion affecting the pons, with a heterogeneous appearance and cystic or necrotic areas (arrow). A gadolinium contrast-enhanced, fat-saturated T1-weighted sequence (B) showing heterogeneous contrast enhancement of the solid portions of the tumor (arrow). The histological findings were consistent with a diagnosis of high-grade glioma, and the molecular evaluation showed that the lesion was IDH wild-type and H3K27-mutated.

**Figure 7:**
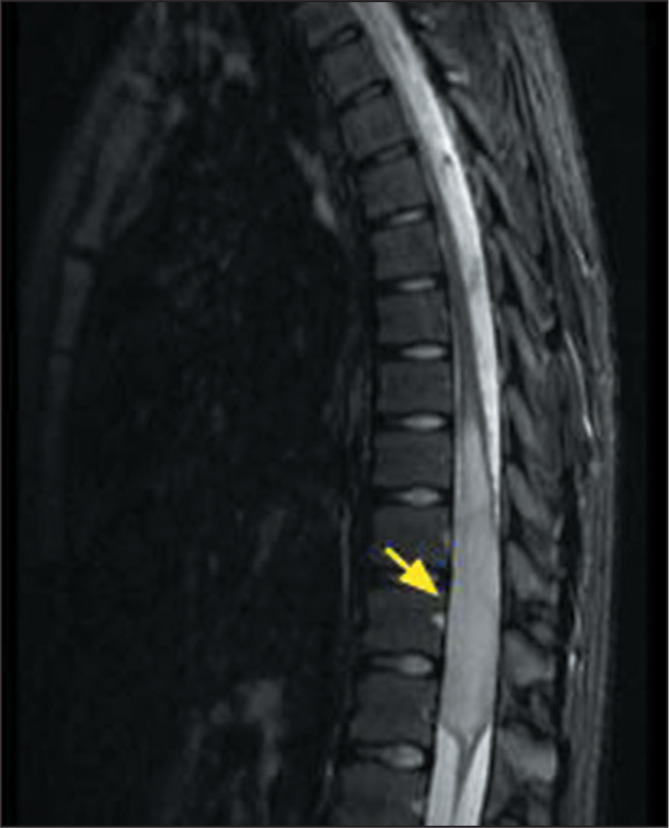
MRI examination of a 12-year-old male patient diagnosed with H3K27-altered diffuse midline glioma. A T2-weighted sequence showing an intra-axial lesion, with high signal intensity, in the spinal cord (arrow). The results of the histological and molecular analyses were consistent with a diagnosis of IDH wild-type, H3K27-altered diffuse midline glioma.

**Figure 8:**
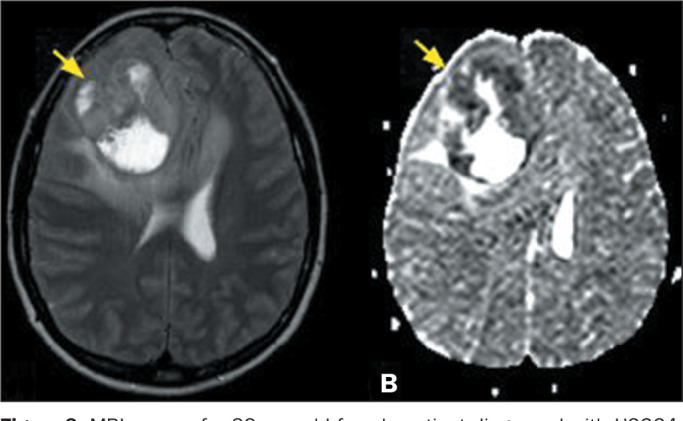
MRI scans of a 32-year-old female patient diagnosed with H3G34-mutant diffuse hemispheric glioma. A T2-weighted sequence (A) showing an expansile, heterogeneous lesion, with an isointense signal delineating central cystic/necrotic areas (arrow), in the right frontal lobe, and a diffusion-weighted sequence (B) showing low apparent diffusion coefficient values in its solid portions (arrow), indicative of restricted diffusion. The histological findings were consistent with a diagnosis of high-grade glioma, and the molecular evaluation showed that the lesion was IDH wild-type and H3G34-mutant.

**Figure 9:**
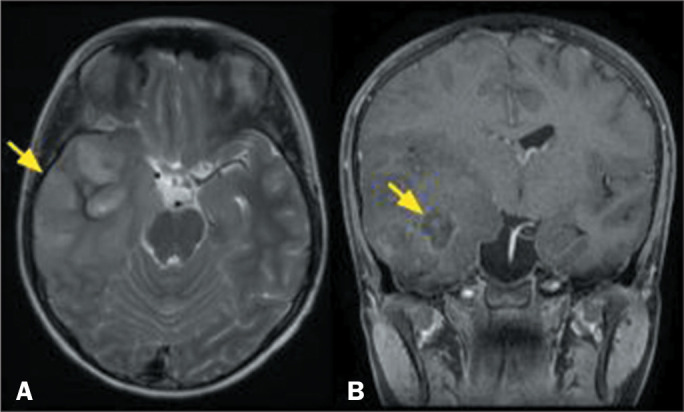
MRI scans of a 11-year-old male patient diagnosed with pediatric-type, H3 wild-type, IDH wild-type high-grade diffuse glioma. A T2-weighted sequence (A) showing an expansile, infiltrative lesion with high signal intensity, in the right temporal lobe (arrow) and a gadolinium contrast-enhanced, fat-saturated T1-weighted sequence (B) showing discrete, heterogeneous contrast enhancement delineating an area without central enhancement, consistent with necrosis (arrow). The histological findings were consistent with a diagnosis of high-grade glial neoplasia, and the molecular evaluation showed that the lesion was H3 wild-type and IDH wild-type, without TERT promoter amplification.

**Figure 10:**
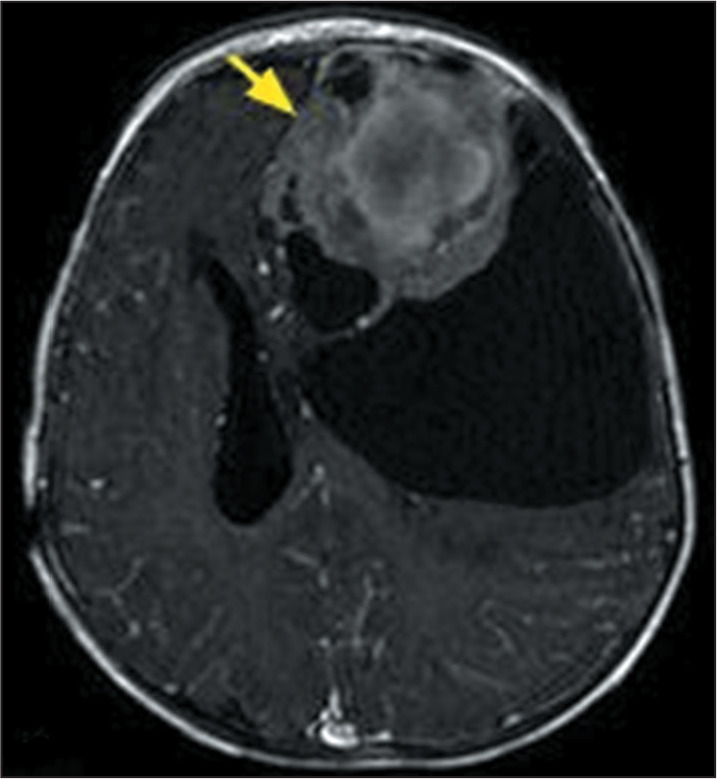
MRI examination of a 11-month-old male patient diagnosed with pediatric-type hemispheric glioma. A gadolinium contrast-enhanced, fat-saturated T1-weighted sequence showing an expansile, heterogeneous lesion in the left frontal lobe, with cystic or necrotic areas and heterogeneous contrast enhancement (arrow). The histological findings were consistent with a diagnosis of high-grade glioma, and a molecular panel revealed neurotrophic tyrosine receptor kinase gene fusion.

Pediatric-type diffuse high-grade gliomas have aggressive behavior and a poor prognosis. This group of tumors includes the following^(3)^: methylation of histone 3 (H3) on lysine 27 (H3K27)-altered diffuse midline glioma (Figures 6 and 7); glycine 34 of H3 (H3G34)-mutant diffuse hemispheric glioma (Figure 8); H3 wild-type, IDH *w*ild-type pediatric-type diffuse high-grade glioma (Figure 9); and pediatric-type hemispheric glioma (Figure 10).

In pediatric-type gliomas, H3 wild-type or IDH wild-type alterations or mutations are more predictive of the risk stratification and prognosis than are histological characteristics alone^(9)^. The most common H3-mutant tumors are H3K27M-altered diffuse midline gliomas, which are most often located in the pons, although they can occur in any midline structure, including the thalamus, hypothalamus, pineal gland, midbrain, vermis cerebellar, and spinal cord^(10)^. They are infiltrative, expansile, poorly defined lesions with high signal intensity on T2-weighted MRI sequences and can include areas of intratumoral necrosis, showing contrast enhancement and high rCBV on T2* perfusion-weighted sequences.

In the 2021 WHO classification, the term glioblastoma refers only to adult-type IDH wild-type diffuse astrocytic glioma and no longer applies to pediatric-type gliomas. The imaging features of H3G34-mutant diffuse hemispheric glioma, pediatric-type H3 wild-type high-grade diffuse glioma, pediatric-type IDH wild-type high-grade diffuse glioma, and pediatric-type hemispheric glioma are similar, not only to those of each other but also to those of adult-type IDH wild-type glioblastoma^(11,12)^, in which the lesion is expansile, infiltrative, and poorly defined, with areas of contrast enhancement and high rCBV, some patients having lesions with cystic or necrotic areas and foci of hemorrhage. It is noteworthy that H3G34-mutant diffuse hemispheric gliomas show marked restricted diffusion in their solid components^(13)^. One important difference that should be borne in mind is the age range; pediatric-type hemispheric glioma is more commonly seen in infants, whereas the others are more commonly seen in children and young adults.

## CONCLUSION

It is important that neuroradiologists be familiar with the new classification of central nervous system tumors for clinical practice, as well as for the evaluation and reporting of imaging examinations of patients with gliomas.
